# Transitions of self-management behaviors and frailty status among community-dwelling older adults: a national longitudinal population-based cohort study

**DOI:** 10.1186/s12877-022-03578-3

**Published:** 2022-11-18

**Authors:** Thi-Lien To, Ching-Pyng Kuo, Chih-Jung Yeh, Wen-Chun Liao, Meng-Chih Lee

**Affiliations:** 1 College of Public Health, China Medical University, Taichung, 406040 Taiwan; 2 Faculty of Nursing, Nguyen Tat Thanh University, Ho Chi Minh, 70000 Vietnam; 3 School of Nursing, Chung Shan Medical University, Taichung, 40201 Taiwan; 4 Department of Nursing, Chung Shan Medical University Hospital, Taichung, 40201 Taiwan; 5 School of Public Health, Chung Shan Medical University, Taichung, 40201 Taiwan; 6 School of Nursing, China Medical University, No. 100, Sec. 1, Jingmao Rd., Beitun Dist., Taichung City, 406040 Taiwan; 7 Department of Nursing, China Medical University Hospital, Taichung, 404332 Taiwan; 8 College of Management, Chaoyang University of Technology, Taichung, 413310 Taiwan; 9 Department of Family Medicine, Taichung Hospital, Ministry of Health and Welfare, No. 199, Sec. 1, San-Min Road, Taichung, 40201 Taiwan

**Keywords:** Frailty, Older adults, Community-dwelling, Self-management behavior

## Abstract

**Background:**

Frailty in older adults is a common geriatric syndrome that could be prevented; thus, coping strategies for the aging population are essential. Self-management behaviors may represent cost-effective strategies to prevent physical frailty in community-dwelling older adults. This study aimed to describe the changes in frailty status among community-dwelling older adults in Taiwan and investigate the association between transitions of self-management behaviors and frailty status over 4 years of follow-up (2007 to 2011).

**Methods:**

Data were retrieved from the Taiwan Longitudinal Study of Aging (TLSA), years 2007 and 2011. In this prospective cohort study, 1283 community-dwelling older adults aged 65 years and older without cognitive impairment were recruited. Frailty was defined based on Fried’s frailty phenotype. Self-management behaviors (maintaining body weight, quitting smoking or no smoking, drinking less or no drinking, exercising, keeping diet control, and maintaining a regular lifestyle) were assessed using a questionnaire. Multinomial logistic regression analyses were used to investigate the associations between changes in self-management behaviors and in frailty status. The age group was further stratified to examine the moderation effect in the relationship between changes in self-management behaviors and in frailty status among older adults.

**Results:**

The prevalence of frailty was 8.7% at baseline and 14.9% after 4 years of follow-up, with 196 (15.3%) deaths. Overall, 514 (40.1%) participants maintained their frailty status, 424 (33.0%) worsened, and only 149 (11.6%) improved. Being aged ≥75 years old, having chronic diseases, and an absence of self-management behaviors were associated with frailty at baseline and after follow-up. Among individuals aged 65–74, compared to those who maintained no self-management behaviors, those who decreased the exercise behaviors (yes-to-no) had a higher risk of worsening (RRR = 2.518), while increasing (no-to-yes) and maintaining (yes-to-yes) frequent physical exercise were associated with a lower risk of worsening (RRR = 0.466 and 0.572, respectively) than stable frailty; those who maintained body weight (yes-to-yes) were associated with a lower risk of worsening (RRR = 0.327) than stable frailty after controlling for individual covariates and chronic diseases. Among individuals over 75 years old, compared to no exerciser, older old who decreased their physical exercise had a higher risk of frailty worsening (RRR = 3.255), and increasing frequent physical exercise (no-to-yes) was associated with an improvement in frailty status (RRR = 3.684). Age was a moderator between the effects of maintaining body weight on frailty worsening. There were no associations between the behavioral transitions of smoking, drinking, diet control, or regular lifestyle on the frailty status changes.

**Conclusions:**

Maintaining body weight and frequent physical exercise increased the ratio of frailty stability among individuals 65–74 years old. Increasing exercise behavior is the only factor to improve their frailty status among older adults aged 75 years and over. Older adults should be encouraged to perform adequate physical exercise and maintain a healthy body weight to maintain the frailty status in younger old aged 65–74 years, and especially perform more frequent exercise to improve frailty status in older old over 75 years.

## Background

Populations throughout the world are aging rapidly. About 14.5% of the population were aged ≥65 years old in 2018, and this rate is predicted to increase to 20.7% by 2026 in Taiwan [1]. Frailty is a clinical syndrome characterized by functional impairment and vulnerability to stressors [2]. It is a precursor to disability [3] and increases the risk of various adverse health outcomes, such as falls, fractures, dependency, hospitalization, and mortality [4]. However, frailty is a dynamic process involving frequent transitions to states of greater or lesser frailty over time [5–7]. The transitions between frailty states were first reported by Gill et al. [5], who assessed 754 community-living adults aged 70 years or older every 18 months over a period of 54 months. Overall, 57.6% of the individuals experienced at least one frailty transition, and participants were more likely to transition to states of greater frailty (43.3%) than lesser frailty (23.0%) [5]. A subsequent meta-analysis of 16 cohort studies that examined frailty transitions reported that 40.6% (95% CI: 36.7–44.7%) of individuals transitioned from non-frail to pre-frail, 18.2% (95% CI: 14.9–21.7%) from prefrail to frail, and only 3.3% (95% CI: 1.6–5.5%) transitioned from frail to non-frail over a mean follow-up of 3.9 years [8]. Therefore, more attention should be paid to the factors associated with the change in frailty status in order to identify measures to prevent the progression of frailty.

A broad range of risk factors is associated with the prevalence of frailty, including sociodemographic, clinical, lifestyle, psychological, and biological factors [9]. Healthy lifestyle behaviors are recognized as factors associated with preventing frailty [10–12]. Multifactorial interventions, including physical exercise, nutrition, psychosocial programs, and cognitive training, effectively improve functional health and frailty among adults over 65 years old [13, 14]. Moreover, previous observations found that underweight, overweight, or obese people were at higher risk of becoming frailty and had a higher mortality risk [12, 15]. Prolonged smoking may also increase the likelihood of frailty and death [16]. In contrast, low-moderate alcohol drinking (≤ 2 drinks per day for men) was associated with improvements in frailty status among frail or pre-frail individuals at baseline [12].

Thus, it is necessary to evaluate the long-term effects of self-management behaviors, including maintaining body weight, quitting smoking or no smoking, drinking less or no drinking, exercising, keeping diet control, and maintaining a regular lifestyle, on changes in frailty status. However, at present, relatively scarce data on the associations between these factors in the prevention of frailty are available. We used a national survey to investigate the relationships between changes in frailty status and in self-management behavior over time with various covariates of individual characteristics. A 4-year national longitudinal population-based cohort study was conducted to describe the changes in frailty status in community-dwelling older adults in Taiwan and investigate the effect of changes in self-management behaviors on changes in frailty status.

## Methods

### Source of data and participants

The Taiwan Longitudinal Study on Aging (TSLA) is a national representative population-based cohort study conducted by the National Institute of Family Planning (1965–2000) and The University of Michigan’s Population Studies Centre (PSC), funded by The National Institute on Aging (NIA) and the Government of Taiwan [17]. The TLSA began in 1989 with follow-up periods of 3 to 4 years. Data are collected from participants aged 60 or older using a self-reported questionnaire through face-to-face interviews, with high response rates of 88.3 to 92% in Wave I to Wave VII. More details of the TLSA sample design and data collection were provided in a previous study [18].

**Fig. 1:**
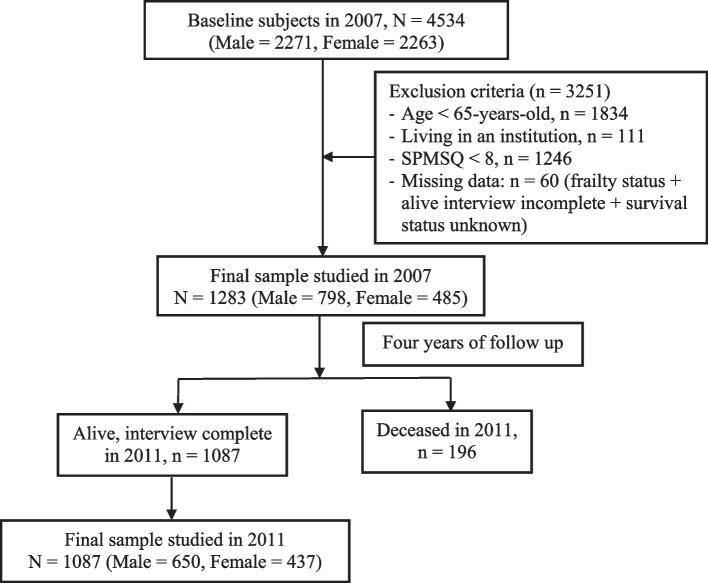
Flow Chart of Study Sample Selection from the Taiwan Longitudinal Study of Aging (TLSA)

This study analyzed data from the cohorts surveyed in 2007 and 2011; details of these cohorts are shown in Fig. 1. Eligible participants were community-dwelling older adults aged 65 years and older. We excluded participants with cognitive impairment (a Short Portable Mental Status Questionnaire score, SPMSQ < 8) [19], living in an institution, or incomplete frailty data in 2007. The study sample comprised 1283 community-dwelling adults aged 65 years or older at baseline (Fig. 1).

### Measures

#### Frailty assessment

Frailty was assessed using the modified versions [20] of Fried’s frailty phenotype [21], which includes five components: shrinking, weakness, exhaustion, slowness, and low physical activity. This modified frailty definition is extensively utilized and has already been validated [20, 22, 23]. Shrinking was considered a lack of appetite that occurred frequently or most of the time in the previous week. For those answering “not applicable” in poor appetite was scored as no shrinking. Weakness was defined as having any difficulty lifting or carrying something weighing 11–12 kg (like two packs of rice). For those answering “not know” in carrying was scored as positive weakness. Exhaustion was considered when subjects answered: “I could not get going” or “I felt everything I did was an effort” often (over 4 days) in the last week. For those answering “not applicable” or “not know” in these two items was scored as positive exhaustion. Individuals who had or were unable to walk 200–300 m were classified as slow. Individuals who did not engage in an outside activity (e.g., walking, hiking, running, or gardening) at least once or twice per week were considered low activity. Frailty scores ranged from 0 to 5 on the five aforementioned items; a higher score indicates poorer frailty status. Respondents who fulfilled at least three of the five items were considered frail; those who met fewer than three criteria were considered non-frail.

#### Change in frailty status

Change in frailty status was calculated by subtracting the baseline score from the 4-year follow-up score, which was then classified into three categories: improvement (< 0), worsening (> 0), and stability (= 0).

#### Self-management behaviors

Participants were asked: “In the past year, have you used some measures in daily life to prevent or control chronic disease?” with six options 1) weight control, 2) quitting smoking or no smoking, 3) drinking less or no drinking, 4) taking regular exercise (≥ 3 times/week and 30 mins/time), 5) eating a healthy diet (a more balanced diet), 6) keeping a regular lifestyle, avoid sitting up for the night, reducing life stress, enough sleep, regular meals… etc. Participants who answered “yes” to the above item were considered positive for that self-management behavior. Changes in self-management behaviors between 2007 and 2011 were classified as 1) maintained no (no to no), 2) decreased (yes to no), increased (no to yes), and maintained yes (yes to yes). Not applicable in smoking or drinking less were considered as no smoking or drinking and classified into the “maintained yes” (yes-to-yes) group in these two behaviors.

#### Personal characteristics

Demographic data included age, gender, education level, marital status, area of residence, and chronic diseases. Age was categorized into two groups: 65–74 years and ≥ 75 years. Education level was classified into lower (illiteracy and elementary school) and higher (junior high school to senior high education and above). The area of residence was categorized into city + urban and rural. Chronic diseases were classified as “yes” and “no” for diabetes mellitus, heart disease, stroke, cancer, lung disease, renal disease, and muscular-skeletal diseases (joint arthritis, hip fracture, and osteoporosis).

### Data analysis

Descriptive statistics for personal characteristics and self-management behaviors are presented as frequencies and percentages for categorical variables. The Chi-squared test was used to compare the distribution of personal characteristics and self-management behaviors between the groups with each frailty status. The association between changes in self-management behaviors and changes in frailty status after follow-up was examined using multinomial logistic regression analyses. The multivariate model included all significant covariates from the univariate analysis and the six self-management behaviors. Frailty stability (no changes in frailty score) and maintained no self-management behavior were set as the reference group in the outcome and independent variables, respectively. The relative risk ratio (RRR) was calculated. In addition, we further stratified the age group (65–74 years versus ≥75 years) to examine the moderation effect of age in the relationship between changes in self-management behavior and in frailty status. Data were processed using SPSS version 26.0 (IBM, Inc., Chicago, IL, USA). The significance level was set at *α* = 0.05; all tests were two-tailed.

## Results

The characteristics of the participants classified by frailty status are shown in Table 1. Almost half of the participants were ≥ 75 years old (46.8%), with a higher proportion of males (62.2%) than females (37.8%). Almost three-quarters (72.3%) of the participants were married, and more than half (60.8%) had only obtained a lower level of education. Of the 1283 respondents, 91.3% (1171) were classified as non-frail and 8.7% (112) as frail at baseline. Older age, female sex, low educational attainment, and a history of heart disease, stroke, lung disease, renal disease, and muscular-skeletal disease were significantly associated with frailty at baseline.

**Table 1:**
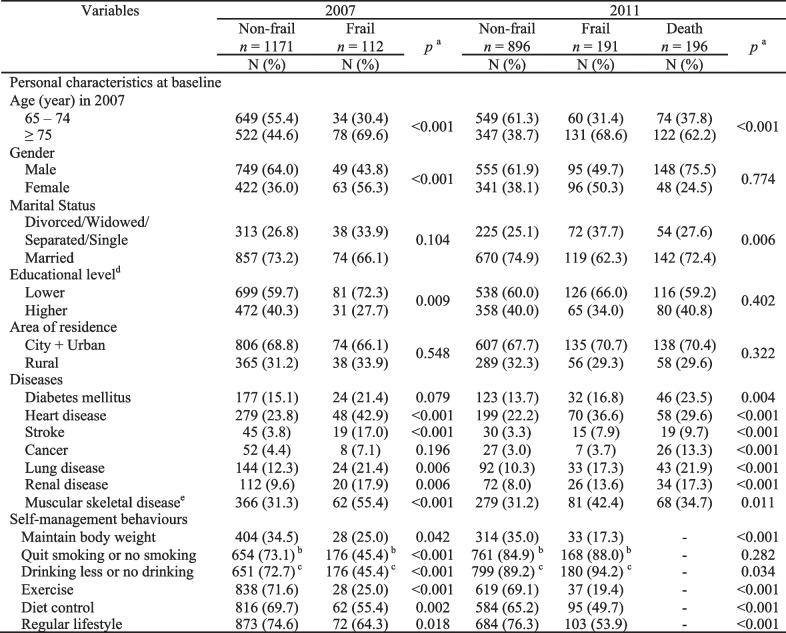
Characteristics of participants on frailty in older adults (*N* = 1283)

^a^ Chi-square tests (two-tailed). In 2011, *p* value was calculated by coding “Death” as frail

^b^ Quit smoking, not applicable/no smoking was coded as “Yes” in 2007 and 2011

^c^ Drinking less, not applicable/no drinking was coded as “Yes” in 2007 and 2011

^d^ Educational level: Lower: illiterate + elementary school, higher: from junior high school to senior high education and above

^e^ Muscular skeletal disease includes joint arthritis, hip fractures, and osteoporosis

During the 4-year follow-up, there were 196 (15.3%) deaths, and 191 (14.9%) participants became frail. Bivariate analysis showed that being over a 75-years-old and having chronic illnesses were associated with a higher risk of being frail (the participants who passed away were classified as frail), whereas being married was associated with a decreased risk of frailty after the 4-year follow-up (Table 1).

Regarding self-management behaviors, the majority of participants maintained a regular lifestyle (73.7%), performed diet control (68.4%), participated in regular exercise (67.5%), quitted smoking or did not smoke (64.7%), and drank less or did not drink (64.5%) (Table 1). Participants who reported maintaining their body weight, exercising, performing diet control, and keeping a regular lifestyle had a lower risk of being frail at baseline and follow-up.

Table 2 shows the transitions of frailty status and self-management behaviors between baseline and after 4 years of follow-up. Overall, 514 (40.1%) participants maintained their frailty status, 424 (33.0%) worsened, and only 149 (11.6%) improved. For changes in self-management behaviors, most of those who maintained behaviors stayed frailty stability.

**Table 2:**
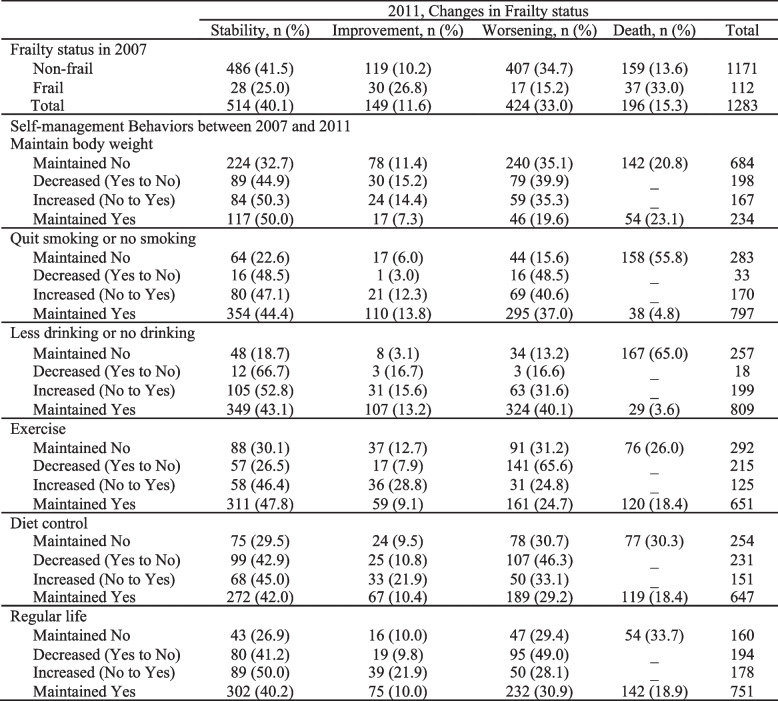
Transitions of frailty status and self-management behaviors between baseline and follow-up

Table 3 presents multinomial logistic regression analyses of the self-management behaviors changes on the frailty status changes between 2007 and 2011, adjusted for personal characteristics and chronic disease. In Model 1 univariate analysis, compared to those who maintained no self-management behaviors, those who maintained (yes-to-yes) or increased their self-management behaviors (no-to-yes) in maintaining body weight, exercise, diet control, and regular lifestyle had a lower risk of frailty worsening rather than frailty stability. Participants who decreased exercise behaviors (yes-to-no) had a higher risk (RRR = 2.39) of frailty worsening, while maintaining frequent exercise (yes-to-yes) was associated with a lower ratio (RRR = 0.451) of improving their frailty status rather than remaining stable. Personal characteristics of those over 75 years, female sex, and a history of heart disease were significantly associated with a higher risk of frailty worsening (RRR = 1.429 ~ 1.689) over frailty stability. In addition, participants with heart disease and muscular-skeletal disease had a higher ratio of frailty improvement than over stability (RRR = 2.373 and 1.717, respectively) (Table 3, Model 1).

**Table 3:**
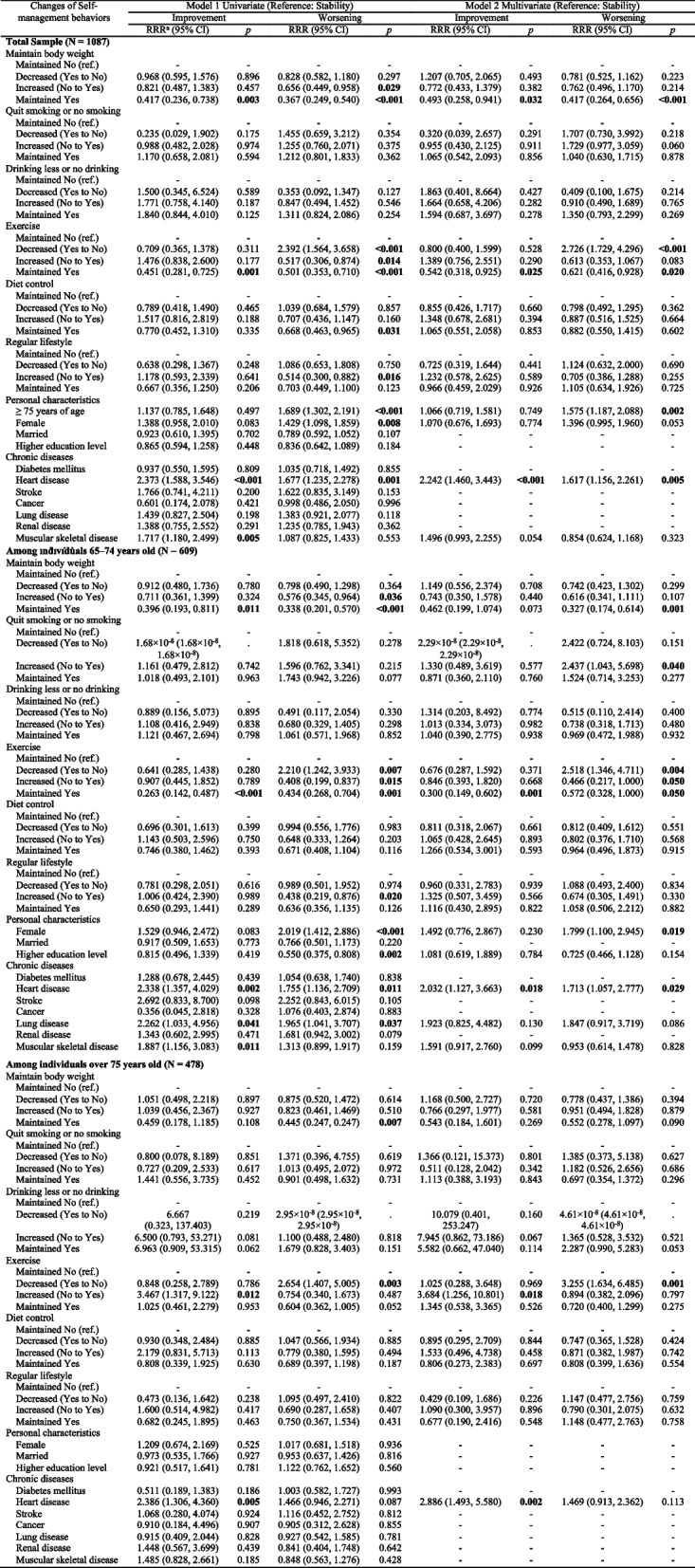
Changes of self-management behaviors on changes in frailty status with age stratification by multinomial logistic regression (*N* = 1087)

^a^ *RRR* Relative Risk Ratio, *CI* confidence interval

Model 1: Univariate multinomial logistic regression

Model 2: Multivariate multinomial logistic regression adjusting for covariates of personal characteristics and chronic diseases

After controlling individual covariates and chronic diseases, the final multivariate multinomial logistic regression model (Table 3, Model 2 Multivariate) showed that decreasing the exercise behaviors (yes-to-no) had a higher risk of worsening in frailty states (RRR = 2.726); maintaining frequent physical exercise (yes-to-yes) predicted a lower risk of worsening (RRR = 0.621) and a lower ratio of improving their frailty status (RRR = 0.542) rather than remaining stable. Participants who maintained body weight (yes-to-yes) had a lower risk of worsening by 58.3% (RRR = 0.417) and a lower ratio of frailty improvement (RRR = 0.493) rather than remaining stable. However, there were no associations between the behavioral transitions of smoking, drinking, diet control, or regular lifestyle on the frailty status changes. Therefore, people who maintained body weight and frequent physical exercise were associated with a higher ratio of frailty stability compared to those who maintained no behaviors. Being aged ≥75 years old remained a prognostic factor for frailty worsening. In addition, a history of heart disease also predicted frailty stability.

Additionally, the moderation analysis stratified by age groups is also shown in Table 3. Age was a moderator between changes in self-management behaviors and in frailty status. In participants 65–74 years old, the risk of maintaining body weight and frequent physical exercise on the frailty worsening were almost similar to the phenomenon of the whole older adults. However, ex-smokers or no smokers had a higher risk of frailty worsening (RRR = 2.437) than stable frailty in the younger old. Adversely, maintaining body weight or quitting smoking behaviors were less likely associated with frailty worsening in people over 75 years. Among individuals over 75 years old, exercise was the only factor affecting their frailty status. Compared to no exerciser, older old who decreased their physical exercise was associated with a higher risk of frailty worsening (RRR = 3.255) as the younger old did. Furthermore, older old with increased frequent physical exercise (no-to-yes) was significantly associated with an improvement in frailty status (RRR = 3.684), which was not significant in the younger old and the whole groups.

## Discussion

This study used national data to describe the changes in frailty status among community-dwelling older adults in Taiwan and investigate the changes in self-management behaviors on the changes in frailty status over 4 years. The prevalence of frailty was 8.7% at baseline and 14.9% after 4 years of follow-up; 15.3% of participants died during follow-up. The prevalence of frailty in our study was similar to reports from Japan (8.7%) [24] and a rural setting in China (8.7%) [25] but slightly higher than analyses conducted in Hong Kong (6.3%) [26], Korea (7.8%) [27], and lower than data from the US (15.3%) [28], European countries (12%) [29], and the estimated global prevalence of frailty (10.7%) [30]. The wide range in the reported prevalence of frailty across these studies may be due to differences in the characteristics of the populations (races, geographical area) and the effect of phenotypic components [30, 31]. However, overall, the prevalence of frailty at baseline in this study is compatible with the rates for other countries.

We examined the changes in frailty status after 4 years of follow-up among community-dwelling older adults. The majority of participants maintained their frailty status (40.1%) (Table 2). In addition, frailty state improvement (11.6%) was higher than previous estimates in Israel (2.1%) [6], Ireland (6%) [32], Italy (8.2%) [12], U.S (8.3%) [33], and China (9.2%) [34], but lower than that reports in Germany (14%) [7], Indonesia (14.8%) [35], 15.1% in Spain [36], Hong Kong (17.9%) [37], Japan (18%) [15], and Malaysia (22.9%) [38]. Transitions to greater frailty are more common than transitions to lesser frailty [5, 10, 33, 39], as we found in this study. The proportion of frailty transition in our study was comparable to a recent meta-analysis study, which revealed that over a mean of 3.9 years, half or more of older adults stayed in the same frailty status, roughly 10% improved, and nearly 40% worsened [8]. Furthermore, our data revealed that the majority of non-frail subjects maintained their status after 4 years of follow-up, which was consistent with previous research in other populations [12, 15, 32, 37, 40]. The wide variations in the follow-up period for each cohort, differences in the regions of study, national income levels, frailty measurements, and the inclusion of mortalities may limit comparisons of the rates of frailty transitions across different studies [8, 41]. Previous studies only assessed the effects of risk factors such as older age and diseases on the reversal of frailty [42]. Thus, the effects of modifiable factors associated with frailty improvement were poorly explored.

Individuals with healthy behavioral habits such as body weight maintenance, exercise, diet control, and regular lifestyle had a lower risk of being frail in our univariate multinomial logistic regression model. The final multinomial logistic regression model revealed that participants aged 65 to 74 who decreased exercise behaviors had a higher risk (2.518) of worsening transition. In contrast, those who maintained regular exercise had a lower risk of frailty worsening as well as a lower ratio of improving their frailty status rather than remaining stable in the latter year after adjusting for personal and disease covariates (Table 3, Model 2). Moreover, maintaining appropriate body weight was also associated with a lower risk of worsening their frailty status rather than remaining stable after adjusting for personal and disease covariates (Table 3, Model 2). These results suggested that among the younger old group, exercise and maintaining appropriate body weight might significantly stabilize frailty status compared to those who maintained no behaviors. Adversely, maintaining body weight was less likely associated with the change in frailty status in people over 75 years. However, increasing physical exercise was associated with a higher ratio (3.684) of improvement in frailty status, and decreasing exercise behavior had a higher risk of frailty worsening (3.255) than stable frailty among the older old group. Currently, exercise with nutrition-based therapies has provided the most evidence for delaying frailty worsening [43]. Our finding was in line with previous studies, which highlighted that low levels of physical exercise elevated the probability of worsening transition [12, 38]. In comparison, performing more regular physical activity reduced probability of frailty worsening [10, 11, 44] and a greater likelihood of frailty improvement [10, 44]. Our results indicate that it is beneficial to encourage seniors aged 65–74 to maintain regular exercise with any type of exercise that may potentially increase the likelihood of frailty state stability and improve their status even aged 75 years and over. Do not give up on exercise, and never start too late. Individualized exercise programs tailored to frailty status and other chronic diseases are necessary to reduce the progression of frailty and prevent adverse outcomes.

Besides, this study also found that maintaining appropriate body weight was associated with a higher ratio of frailty stability among younger old aged 65–74 years after adjusting for personal and disease covariates (Table 3, Model 2), not overweight or underweight. Abdominal obesity has been proven to be associated with greater frailty [35, 45] and mortality [12]. A higher body mass index (BMI) was associated with new-onset frailty [15, 46] and a higher risk of frailty worsening [12]. Increasing BMI also hampered recovering robustness [36, 47]. Excessive weight gain is a major risk factor for metabolic syndrome, negatively affecting cardiovascular systems [48]. In contrast to obesity, being underweight was less likely to worsen frailty but associated with a higher risk of mortality [12]. It is critical to maintain a healthy body weight to avoid frailty status in younger old aged 65–74 years.

In addition, this study found that ex-smokers or never-smokers were associated with a higher risk of frailty worsening in the younger old group, but not in the older old group. This result was different from the study of Pollack et al. [33], that use of tobacco products, either in the past or currently, was a predictor of the progression of frailty [33]. Smoke is a contradictory factor in health, such as cognitive improvement [49]. This cognitive improvement effect of tobacco use may affect younger elders’ frailty status. Further studies are recommended to explore the association between smoking behavior and frailty transition in community-dwelling older adults. The results of multivariate models in this study showed that the behavioral transitions of drinking, diet control, or regular lifestyle were not associated with the changes in frailty status after adjusting covariates. Maintaining body weight and exercise could potentially lessen the likelihood of frailty progression for the younger old, and exercise was the only factor to improve their frailty status in the older old group.

We found that participants aged 75 years or above had an increased risk of frailty worsening (Tables 1 & 3). Previous studies also found that older age increased the risk of frailty worsening [12, 37], and younger age was associated with a higher probability of frailty improvement [37, 42]. As people get older, they gradually undergo age-related degeneration that leads to decreased functional capacity and physiological reserves, making individuals more vulnerable to adverse health outcomes [21, 50]. Thus, it is essential to develop strategies to prevent frailty in high-risk older age groups. The present study found that maintaining body weight, quitting smoking, and exercising are frailty protectors for younger old adults, but only exercise is the protector for older old adults. Age was a moderator in the relationship between the transitions of maintaining body weight and frailty status. We should consider age as a crucial factor when developing the intervention to prevent the progression of frailty and maintain non-frail status in different age groups.

In this study, females were at a higher risk of frailty at baseline (Table 1), which was consistent with other studies [9, 30, 51]; moreover, being female remained a risk factor for change in frailty state in young old aged 65–74, but not in older old over 75 years (Table 3). Compared to men of the same age, women were more likely to be frail due to poorer grip strength, lower average amounts of lean body mass and muscle strength, and increased prevalence of sarcopenia during the aging process [21]. The more steady and progressive decline in females could lead to a higher increase in vulnerability to frailty in women than in males. Men were previously reported to be significantly more likely to maintain non-frail status, while women tend to develop prefrailty [8, 39]. Nevertheless, a recent population-based cohort study of older persons aged 70 and older in Germany indicated that men worsened more frequently than women [7]. Our finding showed that the female gender was the significant prognostic factor for frailty worsening in younger old below 75 years. Lower mortality and higher frailty may lead to a prolonged life span, though with a lower quality of life among females. However, there is no gender difference among older old over 75 years.

Besides, individuals with chronic illnesses including heart disease, stroke, lung disease, and renal disease at baseline were at higher risk of frailty (Table 1), and heart disease remained a factor for the change in frailty status (Table 3, Model 2). These results are consistent with previous studies, which reported that a higher number of comorbidities were significantly associated with frailty [12, 28, 37]. However, in the younger old group, heart disease was associated with a higher ratio of frailty improvement (RRR = 2.032) as well as worsening (RRR = 1.713) rather than remaining stable (Table 3, Model 2). This may indicate that frailty status in patients aged 65–74 with heart disease can be improved or worsened depending on the other factors. The high prevalence of frailty in high-risk populations poses a public health challenge, and efficient strategies must be developed to prevent and reduce the disease burden.

This study was a prospective longitudinal cohort survey of a nationally representative sample. The large sample size and high follow-up rates enabled an accurate estimation of the changes in frailty status after 4 years of follow-up, which could enhance the generalizability of these results among community-dwelling older adults in Taiwan. Notably, this study took into account the changes in self-management behaviors at the baseline and 4 years afterward, using multinomial logistic regression analysis to give a more holistic advice. The current study also explored the moderation effect of age between transitions of self-management behaviors and frailty status and revealed that the beneficial effect of self-management behaviors on changes in frailty status is different among younger old and older old adults. Cognitively impaired individuals (SPMSQ < 8) were excluded to avoid bias due to misreported information. As this cohort of seniors was followed up for 4 years, high attrition rates due to death and missing data were unavoidable. Moreover, this study utilized a secondary database to identify frailty using self-reported measures. This approach may lead to recall bias and misclassification and produce higher frailty estimates than performance-based measures [52]. Self-management behaviors were evaluated using simple questions that are straightforward but less precise than standardized measures, which may lead to under-or overestimation of the actual relationships between self-management behaviors and frailty. We used the Fried frailty phenotype, which may represent a lower prevalence of frailty than other broader phenotypes [30]. Notwithstanding, the Fried frailty phenotype is the most extensively validated scale and has been adopted for various epidemiological cohorts, which could enable comparisons between studies [53]. Finally, because of the study’s design, we do not have frailty information during the period of baseline to follow-up. It is possible that older persons who became frail at the beginning of the study (first 12–24 months) may reduce their physical activity due to frailty. Such a reverse association between exercise and frailty cannot be excluded in this study. Future research to make a more frequent assessment in a yearly manner may ameliorate this limitation.

## Conclusions

This study used national data to investigate the changes in frailty status after a 4-year follow-up among Taiwanese community-dwelling older adults. The prevalence of frailty was 8.7% at baseline and 14.9% after 4 years of follow-up, with 196 (15.3%) deaths. Overall, the majority of the participants maintained or worsened their frailty status; only 11 % of them had improved. For younger old aged 65–74 years who maintained their body weight and kept frequent physical exercise are associated with a higher ratio of frailty stability after controlling for individual covariates and chronic diseases. Among older adults aged 75 years and over, increasing exercise behavior is the only factor to improve their frailty status. There were no associations between the behavioral transitions of smoking, drinking, diet control, or regular lifestyle on the frailty status changes. Older adults should be encouraged to perform adequate physical exercise and maintain a healthy body weight to maintain frailty stability in younger old aged 65–74 years, and especially perform more frequent exercise to improve frailty status in older old over 75 years. Health providers and policymakers should provide insights to help the elderly and caregivers to understand the importance of maintaining body weight and frequent exercise in older adults.

## Data Availability

The datasets used and analyzed during the current study are not publicity available but are available from the Health Promotion Administration at Ministry of Health and Welfare in Taiwan in https://www.hpa.gov.tw/EngPages/Detail.aspx?nodeid=1077&pid=6197 on request with the permission of the Ministry of Health and Welfare, Taiwan.

## Abbreviations

TLSA: Taiwan Longitudinal Study in Aging
SPMSQ: Short Portable Mental Status Questionnaire
RRR: Relative Risk Ratio
CI: confidence interval

## References

[1] National Development Council. Population Projections for the R.O.C. (Taiwan): 2018~2065 2018 [Available from: https://www.ndc.gov.tw/en/cp.aspx?n=2e5dcb04c64512cc.

[2] Morley JE, Vellas B, van Kan GA, Anker SD, Bauer JM, Bernabei R (2013). Frailty consensus: a call to action. J Am Med Dir Assoc.

[3] Kojima G (2017). Frailty as a predictor of disabilities among community-dwelling older people: a systematic review and meta-analysis. Disabil Rehabil.

[4] Vermeiren S, Vella-Azzopardi R, Beckwée D, Habbig A-K, Scafoglieri A, Jansen B (2016). Frailty and the prediction of negative health outcomes: a Meta-analysis. J Am Med Dir Assoc.

[5] Gill TM, Gahbauer EA, Allore HG, Han L (2006). Transitions between frailty states among community-living older persons. Arch Intern Med.

[6] Bentur N, Sternberg SA, Shuldiner J (2016). Frailty transitions in community dwelling older people. Isr Med Assoc J.

[7] Mielke N, Schneider A, Huscher D, Ebert N, Schaeffner E (2022). Gender differences in frailty transition and its prediction in community-dwelling old adults. Sci Rep.

[8] Kojima G, Taniguchi Y, Iliffe S, Jivraj S, Walters K (2019). Transitions between frailty states among community-dwelling older people: a systematic review and meta-analysis. Ageing Res Rev.

[9] Feng Z, Lugtenberg M, Franse C, Fang X, Hu S, Jin C (2017). Risk factors and protective factors associated with incident or increase of frailty among community-dwelling older adults: a systematic review of longitudinal studies. PLoS One.

[10] Ye B, Chen H, Huang L, Ruan Y, Qi S, Guo Y (2020). Changes in frailty among community-dwelling Chinese older adults and its predictors: evidence from a two-year longitudinal study. BMC Geriatr.

[11] Abe T, Nofuji Y, Seino S, Murayama H, Yoshida Y, Tanigaki T (2020). Healthy lifestyle behaviors and transitions in frailty status among independent community-dwelling older adults: the Yabu cohort study. Maturitas.

[12] Trevisan C, Veronese N, Maggi S, Baggio G, Toffanello ED, Zambon S (2017). Factors influencing transitions between frailty states in elderly adults: the Progetto Veneto Anziani longitudinal study. J Am Geriatr Soc.

[13] Ng TP, Feng L, Nyunt MS, Feng L, Niti M, Tan BY (2015). Nutritional, physical, cognitive, and combination interventions and frailty reversal among older adults: a randomized controlled trial. Am J Med.

[14] Seino S, Nishi M, Murayama H, Narita M, Yokoyama Y, Nofuji Y (2017). Effects of a multifactorial intervention comprising resistance exercise, nutritional and psychosocial programs on frailty and functional health in community-dwelling older adults: a randomized, controlled, cross-over trial. Geriatr Gerontol Int.

[15] Doi T, Makizako H, Tsutsumimoto K, Nakakubo S, Kim M-J, Kurita S (2018). Transitional status and modifiable risk of frailty in Japanese older adults: a prospective cohort study. Geriatr Gerontol Int.

[16] Kojima G, Iliffe S, Jivraj S, Liljas A, Walters K (2018). Does current smoking predict future frailty? The English longitudinal study of ageing. Age Ageing.

[17] Bureau of Health Promotion at the Department of Health in Taiwan. Taiwan Longitudinal Study on Aging (TLSA) 2015 [updated 24 September 2019. Available from: https://www.hpa.gov.tw/EngPages/Detail.aspx?nodeid=1077&pid=6197.

[18] Chiao C, Weng LJ, Botticello A (2009). Do older adults become more depressed with age in Taiwan? The role of social position and birth cohort. J Epidemiol Community Health.

[19] Pfeiffer E (1975). A short portable mental status questionnaire for the assessment of organic brain deficit in elderly patients. J Am Geriatr Soc.

[20] Hsu HC, Chang WC (2015). Trajectories of frailty and related factors of the older people in Taiwan. Exp Aging Res.

[21] Fried LP, Tangen CM, Walston J, Newman AB, Hirsch C, Gottdiener J (2001). Frailty in older adults: evidence for a phenotype. J Gerontol A Biol Sci Med Sci.

[22] de Vries NM, Staal JB, van Ravensberg CD, Hobbelen JSM, Olde Rikkert MGM, Nijhuis-van der Sanden MWG (2011). Outcome instruments to measure frailty: a systematic review. Ageing Res Rev.

[23] Chu WM, Ho HE, Yeh CJ, Hsiao YH, Hsu PS, Lee SH (2021). Self-rated health trajectory and frailty among community-dwelling older adults: evidence from the Taiwan longitudinal study on aging (TLSA). BMJ Open.

[24] Murayama H, Kobayashi E, Okamoto S, Fukaya T, Ishizaki T, Liang J (2020). National prevalence of frailty in the older Japanese population: findings from a nationally representative survey. Arch Gerontol Geriatr.

[25] At J, Bryce R, Prina M, Acosta D, Ferri CP, Guerra M (2015). Frailty and the prediction of dependence and mortality in low- and middle-income countries: a 10/66 population-based cohort study. BMC Med.

[26] Yu R, Tang N, Leung J, Woo J (2015). Telomere length is not associated with frailty in older Chinese elderly: cross-sectional and longitudinal analysis. Mech Ageing Dev.

[27] Lee Y, Kim J, Han ES, Ryu M, Cho Y, Chae S (2014). Frailty and body mass index as predictors of 3-year mortality in older adults living in the community. Gerontology.

[28] Bandeen-Roche K, Seplaki CL, Huang J, Buta B, Kalyani RR, Varadhan R (2015). Frailty in older adults: a nationally representative profile in the United States. J Gerontol A Biol Sci Med Sci.

[29] O'Caoimh R, Galluzzo L, Rodriguez-Laso A, Van der Heyden J, Ranhoff AH, Lamprini-Koula M (2018). Prevalence of frailty at population level in European ADVANTAGE joint action member states: a systematic review and meta-analysis. Ann Ist Super Sanita.

[30] Collard RM, Boter H, Schoevers RA, Oude Voshaar RC (2012). Prevalence of frailty in community-dwelling older persons: a systematic review. J Am Geriatr Soc.

[31] Siriwardhana DD, Weerasinghe MC, Rait G, Falcaro M, Scholes S, Walters KR (2019). Prevalence of frailty in rural community-dwelling older adults in Kegalle district of Sri Lanka: a population-based cross-sectional study. BMJ Open.

[32] Romero-Ortuno R, Hartley P, Davis J, Knight SP, Rizzo R, Hernández B (2021). Transitions in frailty phenotype states and components over 8 years: evidence from the Irish longitudinal study on ageing. Arch Gerontol Geriatr.

[33] Pollack LR, Litwack-Harrison S, Cawthon PM, Ensrud K, Lane NE, Barrett-Connor E (2017). Patterns and predictors of frailty transitions in older men: the osteoporotic fractures in men study. J Am Geriatr Soc.

[34] Liu Z-Y, Wei Y-Z, Wei L-Q, Jiang X-Y, Wang X-F, Shi Y (2018). Frailty transitions and types of death in Chinese older adults: a population-based cohort study. Clin Interv Aging.

[35] Setiati S, Laksmi PW, Aryana IGPS, Sunarti S, Widajanti N, Dwipa L (2019). Frailty state among Indonesian elderly: prevalence, associated factors, and frailty state transition. BMC Geriatr.

[36] Rodríguez-Laso Á, García-García FJ, Rodríguez-Mañas L (2022). Transitions between frailty states and its predictors in a cohort of community-dwelling Spaniards. J Am Med Dir Assoc.

[37] Lee JSW, Auyeung T-W, Leung J, Kwok T, Woo J (2014). Transitions in frailty states among community-living older adults and their associated factors. J Am Med Dir Assoc.

[38] Ahmad NS, Hairi NN, Said MA, Kamaruzzaman SB, Choo WY, Hairi F (2018). Prevalence, transitions and factors predicting transition between frailty states among rural community-dwelling older adults in Malaysia. PLoS One.

[39] Lorenzo-López L, López-López R, Maseda A, Buján A, Rodríguez-Villamil JL, Millán-Calenti JC (2019). Changes in frailty status in a community-dwelling cohort of older adults: the VERISAÚDE study. Maturitas.

[40] Li CM, Lin CH, Li CI, Liu CS, Lin WY, Li TC (2021). Frailty status changes are associated with healthcare utilization and subsequent mortality in the elderly population. BMC Public Health.

[41] Ofori-Asenso R, Lee Chin K, Mazidi M, Zomer E, Ilomaki J, Ademi Z (2020). Natural regression of frailty among community-dwelling older adults: a systematic review and Meta-analysis. The Gerontologist.

[42] Kojima G, Taniguchi Y, Iliffe S, Urano T, Walters K (2019). Factors associated with improvement in frailty status defined using the frailty phenotype: a systematic review and Meta-analysis. J Am Med Dir Assoc.

[43] Apóstolo J, Cooke R, Bobrowicz-Campos E, Santana S, Marcucci M, Cano A (2018). Effectiveness of interventions to prevent pre-frailty and frailty progression in older adults: a systematic review. JBI Database System Rev Implement Rep.

[44] Borrat-Besson C, Ryser V-A, Wernli B, Börsch-Supan A, Brandt M, Litwin H, Weber G (2013). 15 transitions between frailty states – a European comparison.

[45] García-Esquinas E, Graciani A, Guallar-Castillón P, López-García E, Rodríguez-Mañas L, Rodríguez-Artalejo F (2015). Diabetes and risk of frailty and its potential mechanisms: a prospective cohort study of older adults. J Am Med Dir Assoc.

[46] Bouillon K, Kivimaki M, Hamer M, Shipley MJ, Akbaraly TN, Tabak A (2013). Diabetes risk factors, diabetes risk algorithms, and the prediction of future frailty: the Whitehall II prospective cohort study. J Am Med Dir Assoc.

[47] Thompson MQ, Theou O, Adams RJ, Tucker GR, Visvanathan R (2018). Frailty state transitions and associated factors in south Australian older adults. Geriatr Gerontol Int.

[48] Flegal KM, Graubard BI, Williamson DF, Gail MH (2007). Cause-specific excess deaths associated with underweight, overweight, and obesity. JAMA.

[49] Campos MW, Serebrisky D, Castaldelli-Maia JM (2016). Smoking and cognition. Curr Drug Abuse Rev.

[50] Clegg A, Young J, Iliffe S, Rikkert MO, Rockwood K (2013). Frailty in elderly people. Lancet.

[51] Gordon EH, Peel NM, Samanta M, Theou O, Howlett SE, Hubbard RE (2017). Sex differences in frailty: a systematic review and meta-analysis. Exp Gerontol.

[52] Theou O, Cann L, Blodgett J, Wallace LM, Brothers TD, Rockwood K (2015). Modifications to the frailty phenotype criteria: systematic review of the current literature and investigation of 262 frailty phenotypes in the survey of health, ageing, and retirement in Europe. Ageing Res Rev.

[53] Bouillon K, Kivimaki M, Hamer M, Sabia S, Fransson EI, Singh-Manoux A (2013). Measures of frailty in population-based studies: an overview. BMC Geriatr.

